# Mechanisms of Motivational Interviewing for Antiretroviral Medication Adherence in People with HIV

**DOI:** 10.1007/s10461-020-02846-w

**Published:** 2020-03-30

**Authors:** Ailbhe Hogan, Delwyn Catley, Kathy Goggin, Michael Evangeli

**Affiliations:** 1 Department of Psychology, Royal Holloway, University of London, Egham, Surrey TW20 0EX UK; 2 Center for Children’s Healthy Lifestyles and Nutrition, Children’s Mercy Kansas City, Kansas City, MO USA; 3 Health Services and Outcomes Research, Children’s Mercy Kansas City, Kansas City, MO USA; 4 School of Medicine, University of Missouri − Kansas City, Kansas City, MO USA

**Keywords:** HIV, Antiretroviral, Adherence, Motivational interviewing, Mechanisms

## Abstract

Antiretroviral therapy (ART) for HIV requires strict regimen adherence. Motivational interviewing (MI) can improve ART adherence. MI process studies have rarely focussed on ART adherence. Such studies may facilitate MI modifications to improve outcomes. This study employed a single group pre and post-test design with 62 adults with HIV (16 female; mean age 40 years). Therapist use of MI-consistent (MICO) methods, MI spirit, and client change and sustain talk were coded from an MI session. Relationships were assessed with ART schedule adherence. MICO methods positively correlated with change and sustain talk and were negatively associated with proportion of change talk. No variables were associated with ART adherence change. Mediation analysis did not support the MI model of change. This may be due to the fact that ART adherence is determined by both motivational and non-motivational factors. It may also be that bidirectional relationships exist between therapist and client speech.

## Introduction

Chronic conditions have long been recognised as the leading causes of death and disability worldwide [1]. Adherence to long-term medication for chronic illnesses, however, is only approximately 50% across conditions [2]. HIV is now considered a chronic condition due to the success of antiretroviral therapy (ART). ART guidelines recommend that all people diagnosed with HIV take ART regardless of CD4 count or viral load [3]. Dose adherence rates of less than 95% are associated with the risk of drug-resistance which can lead to the evolution of drug-resistant strains, progression of the disease, and an increased risk of onward transmission [4, 5]. Schedule adherence (percentage of doses taken on time) is also important [6] to maintain a continuous coverage of ART within the blood to minimise the risk of developing drug resistance. Non-adherence can result in drug-resistant mutations of the virus being transmitted to uninfected people who are then newly infected with a drug-resistant strain of the virus and thus have less effective treatment options available to them [7]. Consequently, adherence to ART medication has public health implications and achieving viral suppression in those receiving ART is one of the goals set out by the UNAIDS – *Lancet* Commission in their plan to end the AIDS epidemic by 2030 [8].

ART adherence can be difficult to achieve and maintain. It is estimated that only 62% of ART users worldwide are achieving adherence rates of at least 90% [9]. A number of barriers to ART adherence have been suggested, including motivational barriers associated with medication and health concerns, and stigma [10]. One promising intervention for enhancing ART adherence is Motivational Interviewing (MI) [11]. Miller and Rollnick state that, “MI is a person-centred counselling style addressing the common problem of ambivalence about change” (page 29) [12]. MI aims to foster behaviour change by eliciting the client’s own motivation for change including by eliciting statements in favour of making a change (‘change talk’) and reducing statements for maintaining the status quo (‘sustain talk’). The therapist aims to accomplish this through embodying ‘MI spirit’ by adopting a collaborative communication style while encouraging client autonomy [12].

Several factors contribute to MI being an appropriate intervention for use with ART adherence. Firstly, MI explicitly focuses on developing a client’s self-efficacy which is recommended as one of the key predictors to target in adherence enhancing interventions [13]. MI has been shown to have larger effects in ethnic minority populations in comparison to non-minority white populations [14]. HIV disproportionately affects certain ethnic minorities such as African-American and Hispanic populations in the US [15] and Black-African populations in the UK [16]. ART adherence is a complex behaviour and may require a multipronged approach to treatment and MI is suitable for integration with other interventions. Finally, MI is recommended as an intervention for enhancing motivation, a key hypothesised determinant of ART adherence [17].

A review of the efficacy of MI for improving medication adherence (mostly for ART) found that MI improves medication adherence when compared to treatment as usual or an educational intervention [18]. A systematic review of MI and ART adherence found that three of the five clinical trials studied reported a significant increase in adherence rates, suggesting that MI holds potential as an intervention to improve ART adherence [19]. An integrative review focussing on people with HIV found promising evidence for MI (16 out of 19 studies demonstrating positive effects for MI), either as a standalone or adjunctive treatment with regards to reducing symptoms of depression, enhancing adherence to ART and reducing risky sexual behaviour [11]. The variation of effect sizes across studies relating to medication adherence [20], however, has prompted investigations into MI mechanisms of change. This may inform adaptations of the intervention across different contexts [21]. Psycholinguistic analyses of sessions form the basis of MI process research. Due to the relational nature of MI both therapist and client responses are key to understanding the processes of change [22].

A hypothesized causal model of MI has been developed [23]. It proposes relational and technical pathways through which behaviour change occurs. The relational component focuses on the therapist conveying MI spirit and empathy. The technical component hypothesises that therapist use of MI-consistent (MICO) methods (such as reflective listening and evoking reasons for change) will elicit and reinforce client change talk, which has been shown to predict behaviour change [24]. Hearing oneself argue for change (change talk) is hypothesised to be causally related to behaviour change in this model [23]. The model proposes that both the relational and technical components can either have a direct or mediated (by client change talk) impact on behaviour change.

A recent meta-analysis of both the relational and technical components of the model has been conducted across multiple peer-reviewed studies targeting alcohol and drug use, gambling, sexual risk behaviour, diet and exercise, and medication adherence (for depression) [25]. There was evidence found in support of the technical hypothesis. A large positive relationship was found between MICO methods and change talk. Contradicting the MI model, a positive medium to large effect was also found between MICO methods and sustain talk. They also studied the proportion of change talk (total change talk/total change and sustain talk) and found a small positive association with the proportion of MICO methods. MI-inconsistent (MIIN) methods (advising, confronting and being directive) were associated with an increase in client sustain talk but not change talk. There was no evidence to support the hypothesised relationship between increased change talk and behaviour outcome. However, the proportion of change talk was found to be positively associated with behaviour change. True to the model there was also a small but significant association found between increased client sustain talk and worse outcomes. The meta-analysis did not find any evidence in support of the relational component of the model.

Given the inconsistent findings relating to the MI model, further process research is needed to gain a better understanding of the causal model of MI. In addition, as there were no MI process studies focussing on medication adherence for chronic physical conditions in the above meta-analysis, it is important to conduct such studies as it is plausible that MI’s mechanisms may differ according to the target behaviour. As MI has a variable effect with ART adherence, examining which within-therapy factors relate to outcome is important to gain a better understanding of the actions of change in this context.

There are only two MI process research studies published with people with HIV. One study explored MI processes within the context of ART adherence [26] namely the relationship between ART adherence and measures of MI session quality and therapist behaviours. The sample (n = 47) was mostly male (79%), of non-white ethnicity (90%), with a mean age of 40 years, and averaged 79% adherence at the end of the trial that data was derived from. A positive association was found for ART adherence and both number of affirming statements made and a higher ratio of reflections to questions asked (indicators of MI-consistent therapist methods). A negative association was found between ART adherence and closed questions (indicator of MIIN therapist methods). Client language – a key aspect of the MI model – and the influence of pre-MI session adherence data was not considered in the analysis. A second study examined if the quality of MI was related to risky sexual behaviour [27]. Participants (n = 32) were mostly male (52%), non-white (84%), with a mean age of 42 years. The behaviour outcome was incidents of unprotected vaginal or anal intercourse in the previous 3 months. The relational pathway was partially upheld as therapist acceptance, MI spirit and empathy were all positively correlated with fewer incidents of unprotected intercourse. Regarding the technical component, only the ratio of therapist reflections to questions was found to be associated with behaviour outcome. The study was limited by the small sample size and the fact that baseline adherence rates and client change language were not measured or controlled for.

This study tested both the relational and technical pathways of the MI model and considered both client and therapist factors in the context of ART adherence. Pre-MI session adherence was controlled for (although the analysis was also carried out using an adherence difference score), and an objective adherence measure was used, along with a larger sample than in previous MI HIV studies. Specifically, this study investigated if client language (change talk, sustain talk and proportion of change talk) mediates the relationship between therapist use of MICO methods and ART adherence change. It also considered if client language (change talk, sustain talk and proportion of change talk) mediates the relationship between therapist MI spirit and ART adherence change.

## Methods

This was a secondary analysis of data collected as part of Project MOTIV8, an RCT exploring the use of MI to increase ART adherence [28]. Participants enrolled in the 48-week research trial (*n* = 204) were randomised to one of three arms: (1) a standard care (SC) group receiving usual medical care (*n* = 65, 32%); (2) an enhanced counselling (EC) group receiving 10 sessions of MI-based cognitive behavioural therapy (CBT) adherence counselling (*n* = 70, 34%) and (3) an enhanced counselling/observed therapy (EC/OT) group receiving 10 sessions of MI-based adherence CBT counselling alongside the supervision of a portion of medication doses (*n* = 69, 34%) for 24 weeks. Ethical approval for the study was first obtained from the appropriate Institutional Review Boards in 2004. Data were collected from December 2004 to August 2009.

### Participants of MOTIV8

Participants were recruited from six outpatient clinics in Kansas City. Eligible participants were HIV positive, over the age of 18, English-speaking, and taking ART for the first time, changing their ART regimen or having self-reported or doctor suspected ART adherence difficulties as evidenced by clinical viral load (HIV RNA > 1,000 copies/ml). Participants were excluded if they lacked the cognitive capacity to consent, were pregnant, did not self-administer their medication, had an acute illness, planned a move that might interfere with participation in the study, or lived outside the specified catchment area.

### Procedure of MOTIV8

Informed consent was obtained for eligible participants interested in taking part. Baseline assessment of demographic, adherence, psychosocial and physical health indicators was collected. Participants were then randomised into one of the three groups. Those randomised for MI-based adherence counselling were scheduled for six one-to-one sessions (baseline, weeks 1, 2, 6, 11, and 23) and four telephone sessions (weeks 4, 9, 15, and 19). Sessions lasted, on average, 25 min. The baseline session consisted of information provision regarding adherence and subsequent sessions used one of 11 skill-building modules (e.g., motivation enhancement, self-monitoring, goal setting, and problem solving). MI for motivation enhancement was always the focus of the week 1 therapy session and consecutive sessions either repeated the MI module or focused on one of the other modules.

### Counsellors

Master’s degree level professionals received training in MI delivered by a licensed clinical psychologist with expertise in MI through a day-long workshop and supervised role-plays. Before delivering therapy, all counsellors were required to demonstrate proficiency in MI skills. All sessions were audio recorded and counsellors received regular weekly supervision from a Motivational Interviewing Network of Trainers (MINT) supervisor, in which random tapes were selected and assessed for fidelity to MI principles using a 26-item measure adapted from another study [29]. 

### Measures of MOTIV8

This secondary analysis includes the following measures:

#### Demographic and Health Information

Baseline demographic information included; age, gender at birth, education level, employment status, sexual orientation and ethnicity. Baseline HIV specific clinical information was gathered and included; CD4 cell count, viral load copies, and if the participant was starting ART for the first time or not.

#### ART Adherence

ART adherence data was collected using an electronic pill-cap known as a Medication Events Monitoring System or MEMS cap. This device captures the date and time when a medication bottle is opened allowing for more accurate assessment of adherence than other methods such as self-report and pharmacy refills [30]. Two measures of ART adherence data were calculated as follows: (1) percentage of prescribed ART doses taken (number of doses taken divided by the number of doses prescribed) and (2) percentage of prescribed ART doses taken on time (within 2 h either side of the scheduled dose time – schedule adherence). Data were calculated at three separate intervals: (1) week 1 (the 7-day period before the first MI session); (2) week 2 (the 7-day period after the first MI session); and week 12 (30 days of adherence data prior to 12th week of the trial). This study focuses on schedule adherence, as it is important to maintain a continuous coverage of ART within the blood to minimise the risk of drug resistance, and only week 1 and 2 adherence data.

#### Motivation to Adhere

A brief self-report measure capturing baseline motivation to adhere to ART was devised for the MOTIV8 study. Participants rated four items using scales from 0 = *not at all* to 10 = *extremely* to rate their need, reasons, readiness, and commitment to adhere strictly to the ART schedule. This measure achieved good internal consistency (α = 0.83).

### Sample Selection

This study focused on coding the first MI counselling session (focused on enhancing motivation and confidence for ART adherence) across both MI conditions of the RCT, given that this was delivered to all of these participants with no evidence of difference in ART adherence across these conditions. In addition, subsequent sessions contained both MI and CBT strategies. Due to the ceiling effect noted in the RCT data whereby participants reported high motivation to adhere to ART, our sample focused mainly on those participants with lower baseline adherence motivation. We reasoned these participants were most appropriate and likely to benefit from the motivation enhancement session offered at week 1. Sixty-six sessions were coded*.* During data analysis it was found that four participants had missing adherence data and they were excluded from the study leaving a final sample of n = 62. Twelve sessions were randomly selected for coding training from the remaining sessions not selected for inclusion in the main analysis for this study.

### Coding Process

The sessions were coded using the Motivational Interviewing Skills Code (MISC) 2.5 [31]. The MISC 2.5 incorporates features of two existing coding frameworks [32, 33] and aims to capture more accurately the subtleties of therapist and client speech. Coding is conducted in a series of three separate coding passes. In the first pass, the coder listens to the session straight through and records global ratings of the therapist on six dimensions using a 5-point Likert scale; acceptance, empathy, direction, autonomy support, collaboration and evocation. MI spirit is derived by calculating the mean value across autonomy support, collaboration and evocation.

In the second pass, the therapy session is parsed into separate speech utterances or thought units [34] so that the individual utterance can be assigned codes. The final pass involves listening to the session and assigning therapist and client utterances a behavioural code as described in the coding manual. Each speech utterance can only be assigned one code.

There are 25 possible codes for therapist language which can be grouped into broader categories. The focus of this study was MICO responses, given its hypothesised causal role. MICO responses are comprised of the following codes; advise with permission, affirm, emphasise control, open question (a question that cannot be answered with a ‘yes’ or ‘no’ response), simple reflections, complex reflections, support, and raise concern with permission. Client language is coded into three broad and mutually exclusive categories; change talk, sustain talk and follow/neutral/ask. Change talk and sustain talk are made up of specific categories of change language either towards or away from change (desire, ability, reason, need, taking steps, other and commitment language) reflecting the client’s current or future state of mind. All other client speech is coded as follow/neutral/ask.

All transcripts were parsed and coded by the first author and queries were resolved through discussion with the final author. An undergraduate psychology student assisted in establishing inter-rater reliability. The MISC 2.5 was used to derive the sum of MI-consistent responses, change talk and sustain talk for each session.

### Coding Training

#### MISC (2.5) Training

The main researcher (AH) spent 30 h in coding training while the research assistant spent 15 h in training. Dr Jon Houck (MISC 2.5 author) provided five sample gold-standard coded transcripts for training purposes. The first author achieved a mean Cohen’s (1960) kappa result of 0.8 (almost perfect agreement) [31] across all three categories of interest (MICO responses, change talk, sustain talk) while the research assistant achieved at least 0.7 (substantial agreement).

### Reliability-Testing

Twelve sessions (22%) were randomly selected for double coding. To prevent coding drift, coding meetings were held following every three sessions coded. Consultation was provided by the final author for any outstanding disagreements.

Cohen’s kappa [35] was used to test the inter-rater agreement for the client and therapist speech as it is commonly used for assessing agreement between categories and is suitable for use with two coders and for fully-crossed designs, where all coders code the data [36]. See Table 1 for the inter-rater reliability estimates for therapist and client speech, indicating substantial agreement [37]. The percentage agreement is also reported for reference.

**Table 1:** Inter-rater reliability of therapist & client speech using MISC 2.5

Variable	Kappa	Percentage agreement
Change talk	.69	92.21
Sustain talk	.65	96.67
Follow/neutral/ask	.71	90.70
MICO responses	.77	91.55

As the global coding section of the MISC 2.5 produces continuous data the inter-rater reliability for MI spirit was calculated using a two-way mixed, absolute agreement, single-measures intra-class correlations (ICC) [38]. The resulting ICC was in the fair range, ICC = 0.53 [39] and is comparable to reliability estimates achieved for MI spirit in other studies [40, 41].

### Data Analyses

Analysis used SPSS 21. Kandall’s tau correlations tested the associations between therapist and client speech. Bootstrapping mediational analyses were conducted using the PROCESS macro V2.16 to investigate the proposed indirect effect of MICO responses and MI spirit on ART schedule adherence as mediated by change talk, sustain talk and by the proportion of change talk. The bootstrapping approach to mediation [42] was taken as it does not assume normality in the data. A simple mediational model allowed for the addition of a covariate, namely pre-session ART schedule adherence data, to account for temporal limitations of mediation analysis. The bias-corrected approach was used to estimate the 95% confidence intervals and 5,000 bootstrapped replications were performed. This analysis was repeated using an ART schedule adherence difference score (week 2 minus week 1) rather than controlling for week 1 schedule adherence.

## Results

### Participants’ Characteristics

Table 2 displays key characteristics of the sample (n = 62). Participants had a mean age of 40 years (range 19–61). More than half (51.6%) identified as either homosexual or bisexual.

**Table 2:** Participant demographic and clinical information (n = 62)

Variable	All participants	
	Mean (SD)	Number (%)
Age, years	40.1 (10.1)	
Male gender at birth		46 (74.2)
Ethnicity		
African-American		33 (53.2)
White		23 (37.1)
Mixed		5 (8.1)
Other		1 (1.6)
Education		
High school degree or less		31 (50.0)
More than high school degree		31 (50.0)
Sexual orientation		
Heterosexual		27 (43.5)
Homosexual		26 (41.9)
Bisexual		6 (9.7)
Other		1 (1.6)
Choose not to answer		2 (3.2)
Employment		
Full-time		9 (14.5)
Part-time		6 (9.7)
Not currently employed		47 (75.8)
Depressive symptoms^a^		
Above clinical threshold		36 (58.1)
First time taking ART		18 (29.0)
Viral Load (copies/ml)—baseline	121,925.5 (156,203.7)	
CD4 count (cells/mm^3^)—baseline	264.1 (177.7)	

^a^Center for Epidemiological Studies Depression Scale (Radloff, 1977)

### Descriptive and Correlational Results

Table 3 displays the descriptive statistics for each variable.

**Table 3:** Descriptive statistics of coding and adherence data

Variable	Median	Interquartile range	Mean	SD
Week 1 (% doses taken)	96.43	71.43–100.00	84.35	22.67
Week 2 (% doses taken)	100.00	76.79–100.00	86.28	25.71
Week 1 (% doses taken on time)	85.71	62.50–100.00	76.05	29.85
Week 2 (% doses taken on time)	89.29	57.14–100.00	74.25	33.49
MI spirit (1–5)	4.00	3.92–4.00	3.88	0.28
Change talk (count)	38.00	30.75–55.75	45.87	24.69
Sustain talk (count)	13.00	8.00–18.25	16.00	12.65
MICO responses (count)	62.50	48.00–76.00	63.95	22.07

As expected, highly significant positive associations were found between MICO responses and both change talk and sustain talk (see Table 4). A significant medium-sized positive correlation was observed between change talk and sustain talk. A significant large negative association was noted between sustain talk and proportion of change talk. A significant medium negative relationship was observed between MICO responses and proportion of change talk. A significant positive correlation was seen between MI spirit and change talk. Week 1 and week 2 schedule adherence were positively correlated with each other, r (60) = 0.59, p < 0.001.

**Table 4:** Kendall’s tau correlation coefficients between change talk, sustain talk, proportion change talk, MICO responses and MI spirit

Variable	1	2	3	4	5
1. MICO responses	–				
2. MI spirit	0.08	–			
3. Change talk	0.37**	0.23*	–		
4. Sustain talk	0.48**	0.02	0.29*	_	
5. Proportion CT	− 0.29*	0.02	0.08	− 0.65**	–

*p = 0.05, **p < .001

### Mediation Analyses

**Fig. 1:**
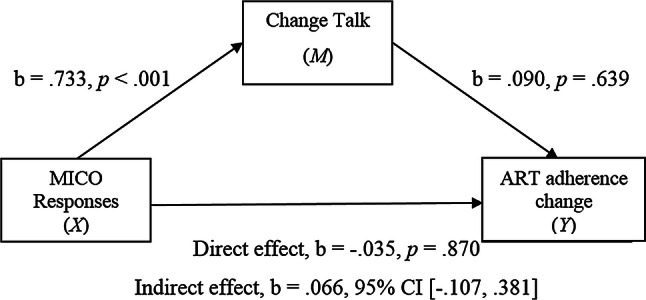
Simple mediation model 1

The first model estimated the indirect effect of an independent variable *X* (MICO responses) on the dependent variable *Y* (ART adherence at week 2) via an intervening or mediating variable *M* (change talk) whilst controlling for ART adherence at week 1 [42]. Figure 1 shows the simple mediational model with the regression coefficients.

As predicted there was a significant positive relationship found between MICO responses and change talk (path *a*). The association between change talk and ART adherence (path *b*) and the direct effect of MICO responses and ART adherence (path *c*) were found to be non-significant. The indirect effect of MICO responses (*X*) on ART adherence (*Y*) via change talk (*M*) was estimated. This is quantified as the product of the regression coefficient estimating path *a* and path *b*. There was no significant indirect effect of MICO responses on ART adherence through change talk.

**Fig. 2:**
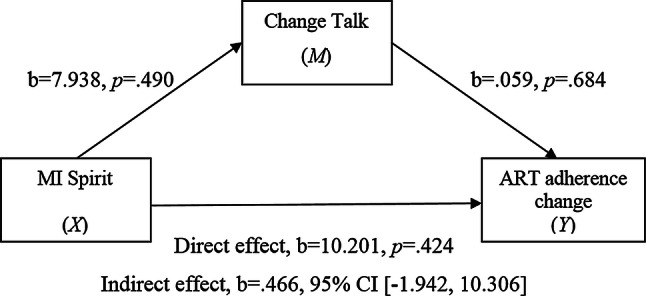
Simple mediational model 2

The second simple mediational model (Fig. 2) estimated the indirect effect of MI spirit (independent variable *X*) on ART adherence change (dependent variable *Y*) through the effect of change talk (mediating variable *M*).

Again, pre-session ART adherence data (week 1) was entered as a covariate. There were no significant associations found between MI spirit and change talk (path a) or between change talk and ART adherence (path b). The direct effect of MI spirit and ART adherence (path c’) was also found to be non-significant. The indirect effect of MI spirit (*X*) on ART adherence change (*Y*) via change talk (*M*) was estimated. There was no significant estimated indirect effect of MI spirit on ART adherence through change talk.

**Fig. 3:**
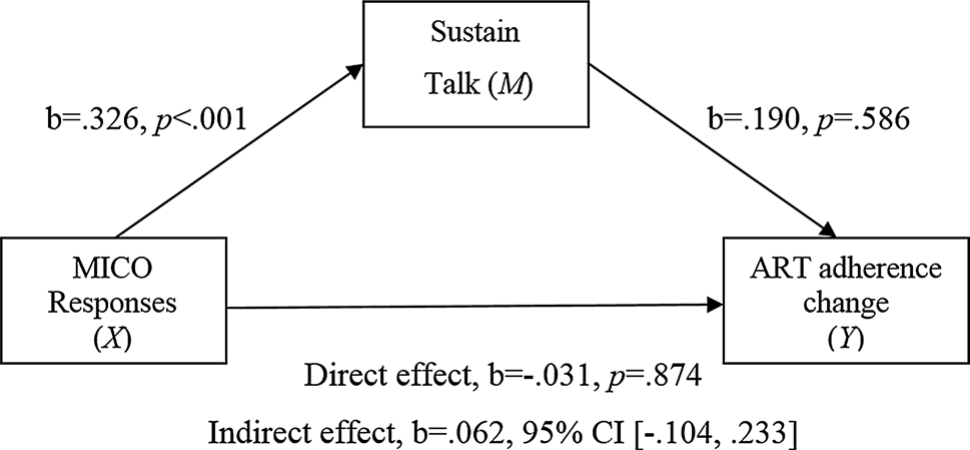
Simple mediation model 3

The third simple mediational model (Fig. 3) estimated the indirect effect of MICO responses on ART adherence change as mediated by sustain talk. Pre-session ART adherence data (week 1) was again entered as a covariate. There was a significant positive relationship found between MICO responses and sustain talk (path *a*). The association between sustain talk and ART adherence (path *b*) and the direct effect of MICO responses and ART adherence (path *c*) were found to be non-significant. There was no significant indirect effect of MICO responses on ART adherence through sustain talk.

**Fig. 4:**
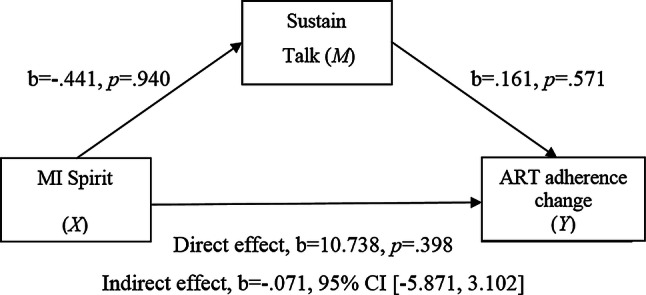
Simple mediation model 4

Figure 4 shows the mediational model testing the indirect effect of MI spirit on ART adherence change (dependent variable *Y*) through the effect of sustain talk. As with change talk there was no significant estimated indirect effect of MI spirit on ART adherence through sustain talk.

**Fig. 5:**
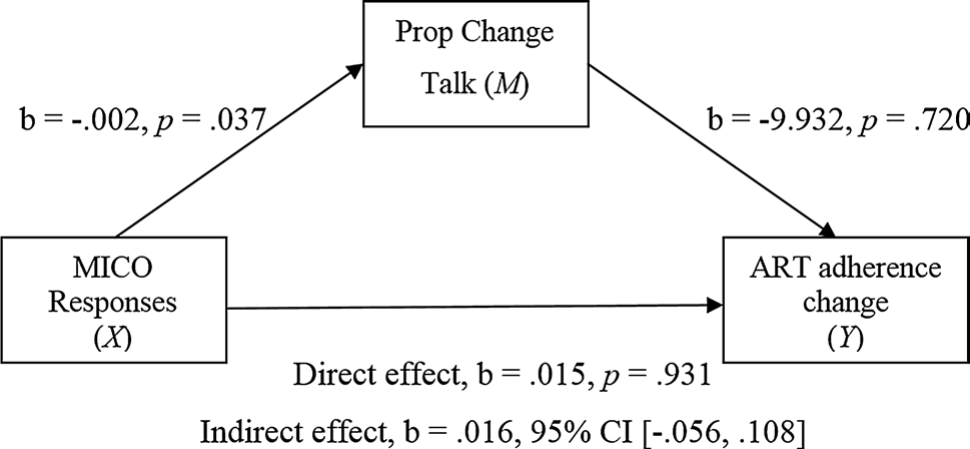
Simple mediation model 5

**Fig. 6:**
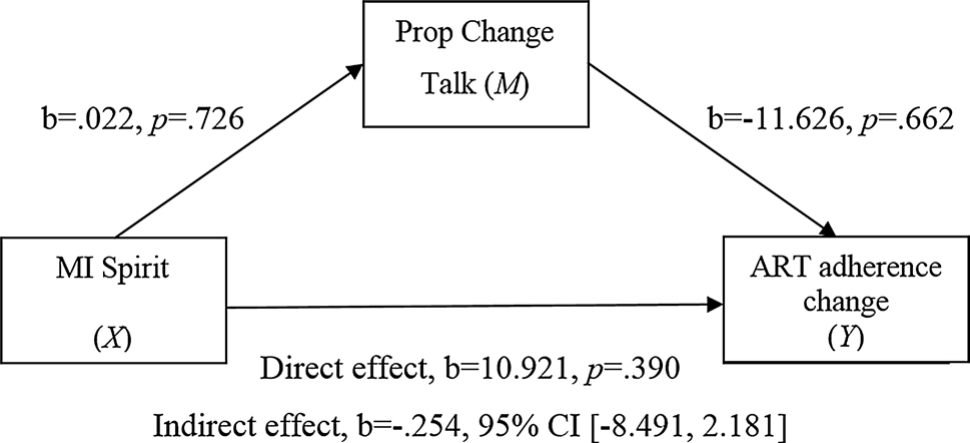
Simple mediation model 6

The above mediation models were also tested with proportion of change talk as a mediator (see Figs. 5 and 6). There were no statistically significant indirect effects detected.

There was, however, a significant negative relationship found between MICO responses and the proportion of change talk (path *a*) (see Fig. 5).

Across all of the mediation analysis, the findings were similar when using an ART schedule adherence difference score.

## Discussion

The aim of the study was to investigate MI mechanisms of change within the context of ART adherence. The causal chain model of MI [23] predicts that both therapist use of MICO methods (technical pathway) and therapist MI spirit (relational pathway) would have an indirect positive effect on improving ART adherence by eliciting client change talk.

Evidence was found in partial support of the technical pathway, with a positive relationship between therapist MICO methods and client change talk, as evidenced in other studies [25]. This relationship may be bidirectional, however, rather than therapist MICO use *causing* client change talk. Sequential analyses (assessing the transition probability of specific therapist speech preceding specific client speech and vice versa) offers additional support for the temporal nature of the mechanism. These have found a stronger association for MICO responses eliciting change talk than vice versa, in the context of substance use [21, 43]. This has not been investigated with medication adherence or in HIV samples.

There was no evidence that change talk mediates the relationship between therapist use of MICO methods and change in ART adherence. This is not consistent with some MI studies with different target behaviours. One study, for example, found evidence that change talk mediated the relationship between therapist use of MICO methods and change in fruit and vegetable consumption [44]. Another found an indirect effect of MICO responses on change in alcoholic drinks per week through client change talk [45]. Unlike the current study both studies demonstrated large changes in target behaviour. In a study with a similar small change in outcome variable (alcohol use in college students), there was no evidence of a mediated relationship [46]. It may have been, therefore, that there was insufficient variability in outcome to show mediated effects or to replicate previously found relationships between sustain talk and outcome, and change talk proportion and outcome [25]. High levels of ART adherence observed prior to the delivery of the MI session (median doses taken on time = 85.71) left little opportunity for improvement in ART adherence. Low variability can influence a correlation [47] and the restricted range in ART adherence change may account for the null findings observed. Alternatively, ART adherence may be a behaviour where there is a considerable influence of behavioural skills (relative to other target behaviours) [17] and, as such, the causal effects of MI mechanisms may be attenuated.

There was a positive association found between MICO methods and client sustain talk. This contrasts with the technical hypothesis which proposes that MICO methods should be associated with reduced sustain talk. Although not explained by the MI model, this association has been observed in meta-analyses and reviews [22, 25, 48] of the MI model which like our study have found that therapist MICO methods is associated with *both* greater change and sustain talk. The therapeutic relationship in MI is built on acceptance and valuing autonomy. Within an MI context, ambivalence is a universal human experience and a normal process on the journey towards change. Therefore, it is not surprising that a therapist displaying MI-consistent skills may elicit and reflect language both towards and away from change, particularly in the initial MI session. Indeed, it may be that proficient therapists are particularly skilled at reflecting sustain talk, which might explain the intriguing finding that a higher frequency of MICO was associated with a lower proportion of client change talk. Again, sequential analysis should be undertaken to examine the temporal nature of these relationships in the context of medication adherence.

The absence of a relationship between therapist use of MICO methods and ART adherence contrasts with research which has found an association between both ratio of reflections to questions and affirming statements and ART adherence, although baseline adherence levels were not controlled for in this research [26]. It is possible that only certain MICO methods such as ratio of reflections to questions may be related to ART adherence change, however the current study focused on an aggregate measure of MICO to limit type I errors.

There was no evidence found in support of the relational pathway of MI within the context of improving ART adherence levels; namely change talk (or sustain talk) was not found to mediate the relationship between MI spirit and ART adherence. This result is consistent with findings reported in a meta-analysis [25], although an association between MI spirit and fewer instances of unprotected anal/vaginal intercourse has been reported in people with HIV [27]. Baseline rates were not controlled for in this study, however. There was little variability in the scores for MI spirit in our study. This may reflect a lack of sensitivity in the scale to detect difference in levels of MI spirit in therapists who are proficient in MI.

This study is limited by the historical nature of the data, the lack of statistical control of potential confounders (e.g., education level) and its predominantly cross-sectional design. We focused on one MI session, where there was no manipulation of counselling process variables and, therefore, it was not possible to establish causality or the timeline of change [49]. It is possible to examine the temporal changes within a session through sequential analysis. As this study was the first test of the technical and relational pathways within the context of medication adherence for any chronic physical condition, a less-resource intensive method than sequential analysis is advised as a first step to establish promising candidates for the MI model in a new population [50]. Future studies should focus on sequential analysis to address the temporality limitation of correlational designs. An additional limitation is that the main study sampling was convenience in nature, rather than representative. Therefore, our findings are best viewed in the context of the specific characteristics of our sample. It may be, for example, that different relationships would have been revealed if participants with even higher levels of baseline ART motivation were included.

It is possible that the varying length of the sessions and the natural verbosity of the therapist or client may have potential confounding effects among behaviour count variables for coding data [51]. It is possible that the positive association found between MICO responses and change talk may be influenced by the length of the session. The fact that MI spirit—a global measure and therefore not as influenced by session length—and change talk were found to be unrelated makes it more likely that session length may have acted as a potential confounding variable. The potential confounding nature of session length and verbosity of speech during the session must be held in mind when interpreting the results of the study.

Another possible explanation for the null findings were the high levels of baseline motivation reported by participants. MI has been developed to target ambivalence and is most effective for people experiencing low levels of motivation for behaviour change [52]. Therefore, the high baseline motivation levels may limit the possibility of MI producing an effect and of showing a potentially mediating role of MI process variables. It is worth noting, however, that there was a good range of frequencies of change talk and sustain talk.

One of the key strengths of this study was the approach taken to the measurement of change in the target behaviour. Pre-session MI adherence levels were measured and were used in the mediational models as either a covariate or to create a difference score. The sample size was larger than other published MI process research studies within the context of HIV-related behaviour change [26, 27]. The sample size was also large enough to make it unlikely that null findings (often associated with small effects) were due to a lack of statistical power.

Before any conclusions can be drawn about the technical and relational components of the MI model [23] within the context of ART adherence it is necessary to repeat the study with a group of people living with HIV who are experiencing low motivation to adhere and low baseline adherence rates. Future studies could also measure how other potential non-motivational adherence determinants (e.g., medication information and behavioural skills) affect the relationships between hypothesised MI mechanisms and outcome. That is, it is acknowledged that motivation (whether or not expressed in client language) is not the only determinant of ART adherence [17].

It is acknowledged that therapist use of MICO methods is a very broad category and future research might focus on exploring the association between different types of MICO responses and client change talk. For example, one study has found that affirming statements made by the therapist is the only MICO response which both increased change talk and decreased sustain talk in the context of reducing hazardous drinking in a student population [53]. A second study has shown that open questions and complex reflections are associated with changes from sustain talk and change talk (and vice versa) compared to simple reflections and paraphrasing [54]. Similarly, aggregate measures of change and sustain talk may contain subtypes of language that are differentially related to outcome following MI [55, 56].

Despite the limitations outlined above this study still found positive associations between therapist MICO methods and client change talk in the context of ART adherence. This relationship appears to be perhaps the most robust aspect of the MI process model. Our study has shown that the lack of support for other aspects of the model now stretches beyond the field of substance use to medication adherence.
